# Medicine and Dentistry Working Side by Side to Improve Global Health Equity

**DOI:** 10.1177/00220345221088237

**Published:** 2022-03-24

**Authors:** F. Lobbezoo, G. Aarab

**Affiliations:** 1 Department of Orofacial Pain and Dysfunction, Academic Centre for Dentistry Amsterdam (ACTA), University of Amsterdam and Vrije Universiteit Amsterdam, Amsterdam, The Netherlands

**Keywords:** intersectoral collaboration, health education, global health, oral health, health workforce, delivery of health care

## Medicine and Dentistry: Worlds Apart

Throughout history, medicine and dentistry have followed their own distinct modi operandi. Unfortunately, this separation has had a detrimental impact upon both individual patients and society at large, insofar as it has exacerbated the poor oral health of many people, in terms of cavities and periodontal disease (Peres et al. 2019). This is especially the case among vulnerable sections of the population, such as those from low-income backgrounds, older people, people with disabilities and chronic diseases, those who have limited access to oral health care, and those who lack oral health insurance coverage (Peres et al. 2019; Simon and Giannobile 2021). The ensuing economic burden hereof is enormous. Indeed, in 2015, the direct and indirect costs associated with poor oral health were estimated at around 545 billion USD worldwide (World Health Organization [WHO] 2021). This is comparable to the economic burden associated with the 2 most expensive illnesses, namely, cardiovascular diseases and diabetes (WHO 2021). Moreover, at the level of the individual patient, poor oral health has also been found to have a notable impact. This is because conditions like cavities and periodontal disease are associated, among other things, with oral discomfort, orofacial pain, tooth loss, reduced chewing ability, poor orofacial aesthetics, and bad breath (Peres et al. 2019). All of these, in turn, can lead to impaired oral health–related quality of life, shortfalls in learning at school, productivity losses at work, and even family disruption (Peres et al. 2019).

## The Mouth: An Integral Part of the Digestive and Respiratory Tract

The problematic yet ongoing separation between general health care managed by medical doctors and oral health care managed by dentists is all the more surprising given that the mouth is undoubtedly both an inseparable and integral part of the digestive and respiratory tract. This testifies to the naturally existing, strong association between both disciplines and professions. In light of this connection, the WHO recently recommended that all of its member states address all of the implications stemming from the insight that oral health should be considered an integral part of general health and find ways to action this within their respective health care systems (WHO 2021). This WHO recommendation is grounded in the nascent awareness that poor oral health is a major contributor to general health conditions (WHO 2021). Notably, oral diseases have increasingly been associated with some of the most prevalent noncommunicable diseases, namely, cardiovascular diseases, diabetes, and cancers (Seitz et al. 2019). These diseases share common risks, including social determinants (e.g., low income, low level of education) and associated risk behaviors (e.g., frequent smoking, high levels of sugar consumption) (Seitz et al. 2019). Alongside this, there is an emergent evidence base for the associations between poor oral health and a host of other conditions, such as respiratory diseases, sleep-related breathing disorders, and cognitive decline (Seitz et al. 2019). This testifies to the tremendous importance of the recent WHO call to strengthen cross-sectoral collaboration between general and oral health (WHO 2021).

## Physicians Reaching Out to Dentists

The aforementioned connections between poor oral health and general health conditions require an adequate level of essential, evidence-based, and bidirectional expertise and skill sharing between medical doctors and dentists. In that context, it is noteworthy to mention that increased attention is being paid to the importance of medical doctors carrying out oral health assessments on patients who lack straightforward and/or regular access to oral health care and prevention (Lee and Somerman 2018). In order to mobilize general health care staff to improve oral health, health care systems have to undergo serious reform. In addition, national and global health care planning, politics, and legislation should be aligned with new medical curricula across all levels of study (i.e., undergraduate, graduate, and postgraduate) worldwide. Dental schools and the dental profession as a whole should reach out to help the medical discipline and profession in this process. Crucially, in order to enable such integration, medicine and dentistry must be willing to abandon their prevailing silo approach to health care (i.e., interprofessional competition). Doing so is in the best interests of patients, insofar as it will ultimately engender greater health equity.

## Dentists Reaching Out to Physicians

With regard to the opposite end of the spectrum, that is, essential expertise and skill sharing between medical doctors and dentists, research has shown that dentists consider medical screening to be important (Greenberg et al. 2010), particularly for cardiovascular diseases, diabetes, and infectious diseases, such as hepatitis, human immunodeficiency virus infection, and—more recently—severe acute respiratory syndrome coronavirus 2 infection. In addition, dentists are ready and willing to incorporate this into their everyday practices (Greenberg et al. 2010). Hence, all of the preconditions appear to be in place to enable oral health care professionals to play a vital role in the early recognition and, importantly, prevention of some of the major noncommunicable and infectious diseases. Hence, in accordance with the brief oral health assessment that has been advocated for medical professionals, additional research into both the required content and feasibility of a basic general health assessment tool for oral health care professionals should urgently be promoted and carried out. Evidently, dentists should organize their medical assessments and general preventive activities in close collaboration with medical doctors. In this respect, a silo mentality is wholly undesirable and can only produce detrimental outcomes for patients. In addition, local traditions, politics, and legislation will ultimately determine the framework within which dentists can assume a prominent role in the early recognition and prevention of medical conditions. Moreover, developing and implementing electronic health records systems that integrate both dental and medical patient information will be beneficial to this end. Such systems should allow for seamless and secure transit of patient information between medical schools/hospitals and dental schools/clinics within the same institution or eventually even across diverse practice settings within the same community. Finally, improving the medical training provided to dentists at the undergraduate, graduate, and postgraduate levels is of paramount importance if this extended role for oral health care professionals is to be successful.

## Medicine and Dentistry: Worlds United

It is recommended that medicine and dentistry evolve beyond their prevailing worlds-apart modi operandi and instead embrace an urgently needed worlds-united approach. To that end, health care planning, politics, and legislation should be aligned with new, partially overlapping medical and dental curricula worldwide. Only then will medical doctors and dentists be able to work sustainably together in research, education, prevention, and care provision, for the express aim of improving health equity around the globe.

## Author Contributions

F. Lobbezoo, contributed to conception, drafted the manuscript; G. Aarab, contributed to conception, critically revised the manuscript. Both authors gave final approval and agree to be accountable for all aspects of the work.
